# Association between thyroid dysfunction and prognosis in patients with liver failure: a systematic review and meta-analysis

**DOI:** 10.3389/fendo.2025.1748907

**Published:** 2026-01-09

**Authors:** Shaoyin Bao, Yin Pan, Yanyun Ruan, Hozeifa Mohamed Hassan, Hongsheng Lu, Qi Chen

**Affiliations:** 1 Precision Medicine Center, Taizhou Central Hospital (Taizhou University Hospital), Taizhou, China; 2 Department of Surgical Oncology, Taizhou Central Hospital (Taizhou University Hospital), Taizhou, China; 3 Department of Pathology, Taizhou Central Hospital (Taizhou University Hospital), Taizhou, China

**Keywords:** free triiodothyronine, liver failure, meta-analysis, mortality, thyroid dysfunction

## Abstract

**Objective:**

To systematically evaluate the association between thyroid dysfunction and prognosis in patients with liver failure.

**Methods:**

We systematically searched PubMed, Embase, Web of Science (WOS), and China National Knowledge Infrastructure (CNKI) till August 2025 to identify prospective or retrospective studies assessing the relationship between thyroid function—specifically, thyroid-stimulating hormone (TSH), free triiodothyronine (FT3), and free thyroxine (FT4)—and outcomes in adult patients with liver failure, including mortality, intensive care unit (ICU) admission, and organ failure. Study quality was evaluated using the Newcastle–Ottawa Scale (NOS). Pooled relative risks (RRs) with 95% confidence intervals (CIs) were calculated, and heterogeneity, subgroup, and sensitivity analyses were conducted.

**Results:**

Eleven studies, including 3,595 patients from four countries, were included. Thyroid dysfunction was significantly associated with increased mortality in patients with liver failure (RR = 3.56, 95% CI: 2.77–4.57; *I²* = 36%). Subgroup analyses by sex, FT3 cut-off, and etiology (hepatitis B virus [HBV] vs. non-hepatitis B virus acute-on-chronic liver failure [non-HBV ACLF]) showed consistent associations. Additionally, a subgroup analysis comparing FT3 concentrations between survival and death groups in liver failure patients revealed a significant difference in FT3 levels between the two groups (mean difference [MD] = 0.89 [0.41, 1.37] for short-term mortality and MD = 0.42 [0.23, 0.61] for long-term mortality). Sensitivity analyses confirmed the robustness of the results. No substantial publication bias was observed.

**Conclusions:**

Thyroid dysfunction, particularly low FT3 levels, is associated with higher mortality rates in patients with liver failure. These findings suggest that routine thyroid function assessment may help identify high-risk patients. Further well-designed prospective studies are needed to clarify the underlying mechanisms and evaluate the potential therapeutic implications.

## 1 Introduction

Liver failure is a severe clinical condition characterized by sudden deterioration of hepatic function, systemic inflammatory activation, and progression to multiple organ dysfunction (1, 2). Despite substantial advances in intensive care and liver transplantation, the mortality rate remains extremely high (3). Accurate prognostic assessment is essential for clinical decision-making and resource allocation. However, the currently available scoring systems, such as the Model for End-Stage Liver Disease (MELD) and the Chronic Liver Failure–Sequential Organ Failure Assessment (CLIF-SOFA), exhibit limited predictive accuracy (4, 5). The identification of novel prognostic biomarkers is therefore of considerable importance.

The thyroid-liver axis is pivotal for systemic metabolic regulation: the liver mediates thyroid hormone deiodination, conjugation, and clearance, while thyroid hormones regulate hepatic lipid, glucose, and protein metabolism (6–8). In liver failure, this axis is disrupted, most notably resulting in non-thyroidal illness syndrome (NTIS), a condition characterized by abnormal thyroid function tests in the absence of primary thyroid disease, including low free triiodothyronine (FT3), normal or low free thyroxine (FT4), and normal thyroid-stimulating hormone (TSH) (9, 10). This syndrome differs from primary hypothyroidism (where TSH is elevated) and is recognized as a marker of systemic illness severity.

The distinct trajectories of thyroid hormones in liver failure are mechanistically driven: FT3 declines early and consistently from​ reduced hepatic type 1 deiodinase (D1) activity and impaired peripheral conversion of thyroxine (T4) to FT3, a process exacerbated by systemic inflammation (11, 12); FT4 typically remains normal or mildly decreased, as hepatic clearance of FT4 is partially preserved and binding to thyroid-binding globulin is altered but not abolished (9, 12); TSH is usually within the normal range, reflecting intact hypothalamic-pituitary feedback, which is a critical distinction between NTIS and central hypothyroidism (13). These alterations have been linked to metabolic disturbances, immune dysfunction, and multi-organ failure progression.

A growing body of research has explored thyroid dysfunction as a prognostic factor in liver failure, but critical gaps persist: several studies report low FT3 as a prognostic marker for mortality (14), while others show varying or inconclusive findings (15, 16). These discrepancies may stem from small sample sizes, heterogeneous definitions of thyroid dysfunction (FT3-based vs. TSH-based), variable etiological spectra (HBV-related vs. non-HBV liver failure), and differing follow-up durations.

Given these uncertainties, a more robust synthesis of available data is necessary. To ensure methodological consistency and comparability of prognostic outcomes across studies, we focused specifically on cohort designs. Cohort studies provide a clear temporal sequence between thyroid dysfunction and subsequent clinical outcomes and are generally considered more reliable for estimating prognostic associations. To address the limitations of existing evidence, we performed a systematic review and meta-analysis of cohort studies to investigate the relationship between thyroid dysfunction and prognosis in patients with liver failure. Subgroup analyses were also carried out to examine the influence of sex, FT3 cut-off values, underlying etiologies, and the association between FT3 concentrations and survival outcomes in patients with liver failure, specifically comparing the FT3 concentrations between survival and death groups. Clinically, this work may complement existing prognostic tools by helping to identify high-risk patients, guide closer monitoring, and support the design of future interventional studies, with the ultimate goal of improving outcomes in this high-mortality population.

## 2 Methods

### 2.1 Literature search

A systematic literature search was conducted in the PubMed, Embase, Web of Science (WOS), and China National Knowledge Infrastructure (CNKI) databases til August 2025. The objective of this search was to identify studies that assess the association between thyroid function and prognosis in liver failure patients. Thyroid function was defined by levels of TSH, FT3, FT4, and related hormones, with a focus on prognostic outcomes including mortality, intensive care unit (ICU) admission, and organ failure.

The keywords covered terms related to liver failure, thyroid function and hormones, and prognosis or risk factors. Terms from different categories were combined using the Boolean operator AND, whereas terms within the same category were combined with OR. Searches were limited to English-language studies (Chinese allowed for CNKI) and adult populations only. The reference lists of relevant reviews were manually screened to identify additional eligible studies. Potential bias from non-English literature exclusion: Non-English studies outside CNKI were excluded due to limited access to language-specific databases.

Titles and abstracts were independently screened by two reviewers, and full texts were assessed for eligibility. Disagreements regarding inclusion were resolved through discussion. The detailed electronic search strategy is presented in the Supplementary File.

### 2.2 Inclusion and exclusion criteria

This study followed the Preferred Reporting Items for Systematic Reviews and Meta-Analyses (PRISMA) guidelines for systematic reviews and meta-analyses (17).

Studies were included if they met all of the following conditions (1): prospective or retrospective cohort design (2); adult patients diagnosed with liver failure according to accepted clinical criteria (3); thyroid function assessed at baseline or during hospitalization using FT3, FT4, TSH, or their predefined categories; and (4) reported at least one prognostic outcome (mortality, ICU admission, organ failure) together with effect estimates or extractable data. The exclusion criteria were as follows (1): non-original studies, such as reviews, meta-analyses, conference abstracts, editorials, letters, or commentaries (2); studies involving participants under 18 years of age, pregnant women, or pediatric/adolescent populations (3); eligible studies that included prospective and retrospective cohort studies; case-control and cross-sectional studies were excluded (4); studies including patients with malignancy or severe infections, or those that did not report risk estimates, such as odds ratios (OR) or relative risks (RR), for thyroid function or hormone levels in relation to liver failure outcomes; and (5) studies that reported thyroid function in formats that could not be harmonized and for which effect estimates were not obtainable.

Duplicate records were removed, and titles and abstracts were screened to exclude irrelevant studies. The remaining articles were assessed through full-text review based on the inclusion and exclusion criteria. Two independent investigators (Shaoyin Bao and Hozeifa M. Hassan) evaluated all eligible studies, and any disagreements were resolved by discussion with a third investigator (Qi Chen).

### 2.3 Data extraction and quality assessment

Two investigators (Shaoyin Bao and Hozeifa M. Hassan) independently extracted data from the included studies. The following information was collected: first author, year of publication, country or region, study design, sample size, baseline characteristics of participants (age and sex distribution), type of liver failure, type and definition of thyroid dysfunction, follow-up duration, primary outcomes (mortality, ICU admission, and incidence of organ failure), and effect estimates (OR and RR with 95% CI). When effect sizes were not directly reported, they were calculated from available data. Additionally, a standardization of serum FT3 and FT4 concentrations to pmol/L was performed to ensure comparability across studies.

The quality of included cohort studies was assessed using the Newcastle–Ottawa Scale (NOS), which evaluates participant selection, comparability of groups, and outcome assessment. Full details of the scoring process are provided in the Supplementary File. The scale ranges from 0 to 9 points, with a score of >7 considered high quality. Data extraction and quality assessment were conducted independently by two reviewers, and any discrepancies were resolved through discussion with a third investigator (Qi Chen).

### 2.4 Statistical analysis

All retrieved records were imported into EndNote (version 21, Clarivate Analytics, Philadelphia, PA, USA https://endnote.com) for reference management. Duplicate publications were initially removed using EndNote’s automated deduplication function and subsequently checked manually to ensure accuracy. All statistical analyses were performed using RevMan (Review Manager version 5.4, Cochrane Collaboration, Copenhagen, Denmark https://training.cochrane.org/online-learning/core-software/revman) and R software (version 4.4.1, R Foundation for Statistical Computing, Vienna, Austria https://www.r-project.org). The relative risk (RR) was used to assess the association between thyroid dysfunction and liver failure outcomes. The mean difference (MD) was applied to compare the average differences in continuous outcomes between patient groups.​​ The 95% confidence interval (CI) indicated the precision of estimates. The *P*-value determined statistical significance, while the Chi-squared (Chi²) test assessed heterogeneity, and the degrees of freedom (df) were calculated based on the analysis categories. The Z-score was reported to evaluate the strength of the effect. RRs with corresponding 95% CIs were calculated as the pooled effect size. Heterogeneity across studies was assessed using the Cochran’s Q test and quantified by the *I²* statistic. An *I²* > 50% or *P* < 0.10 for the Q test was considered indicative of substantial heterogeneity, in which case a random-effects model was applied; otherwise, a fixed-effects model was used.

Analyses were conducted at both the overall population level and across several predefined subgroups (1): sex-based subgroup analysis (male vs. female), which was performed using sex-stratified data extracted from the included studies. To reduce ecological bias, analyses were based on within-study sex-specific comparisons, and studies without sex-stratified data were excluded from subgroup pooling (2); cut-off subgroup analysis, categorized as Low cut-off subgroup (FT3 ≤ 2.8 pmol/L, the established​ cut-off value derived from a previously published study (18)) and High cut-off subgroup (FT3 > 2.8 pmol/L), restricted to acute-on-chronic liver failure (ACLF) patients; and (3) liver failure etiology subgroup analysis, limited to hepatitis B virus (HBV)-related ACLF and ACLF of other causes due to sample size constraints; and (4) subgroup analysis comparing FT3 concentrations between survival and death groups in liver failure patients.

Sensitivity analyses were performed in two ways (1): by sequentially omitting one study at a time (leave-one-out method) to assess the stability of pooled results and (2) by excluding studies rated as low quality (NOS score ≤ 7); and (3) a pre-planned sensitivity analysis was conducted to address heterogeneity in the definition of thyroid dysfunction. As reduced TSH reflects hypothalamic–pituitary axis dysfunction rather than the peripheral low FT3 pattern typical of NTIS in liver failure, studies defining thyroid dysfunction solely by TSH were excluded from the primary meta-analysis to maintain clinical and pathophysiological consistency.

Publication bias was evaluated when ≥ 10 studies were included. Funnel plots were visually inspected, and both Egger’s regression test and Begg’s rank correlation test were conducted using R software. A two-tailed *P* < 0.05 was considered statistically significant.

## 3 Results

### 3.1 Study selection

**Figure 1:**
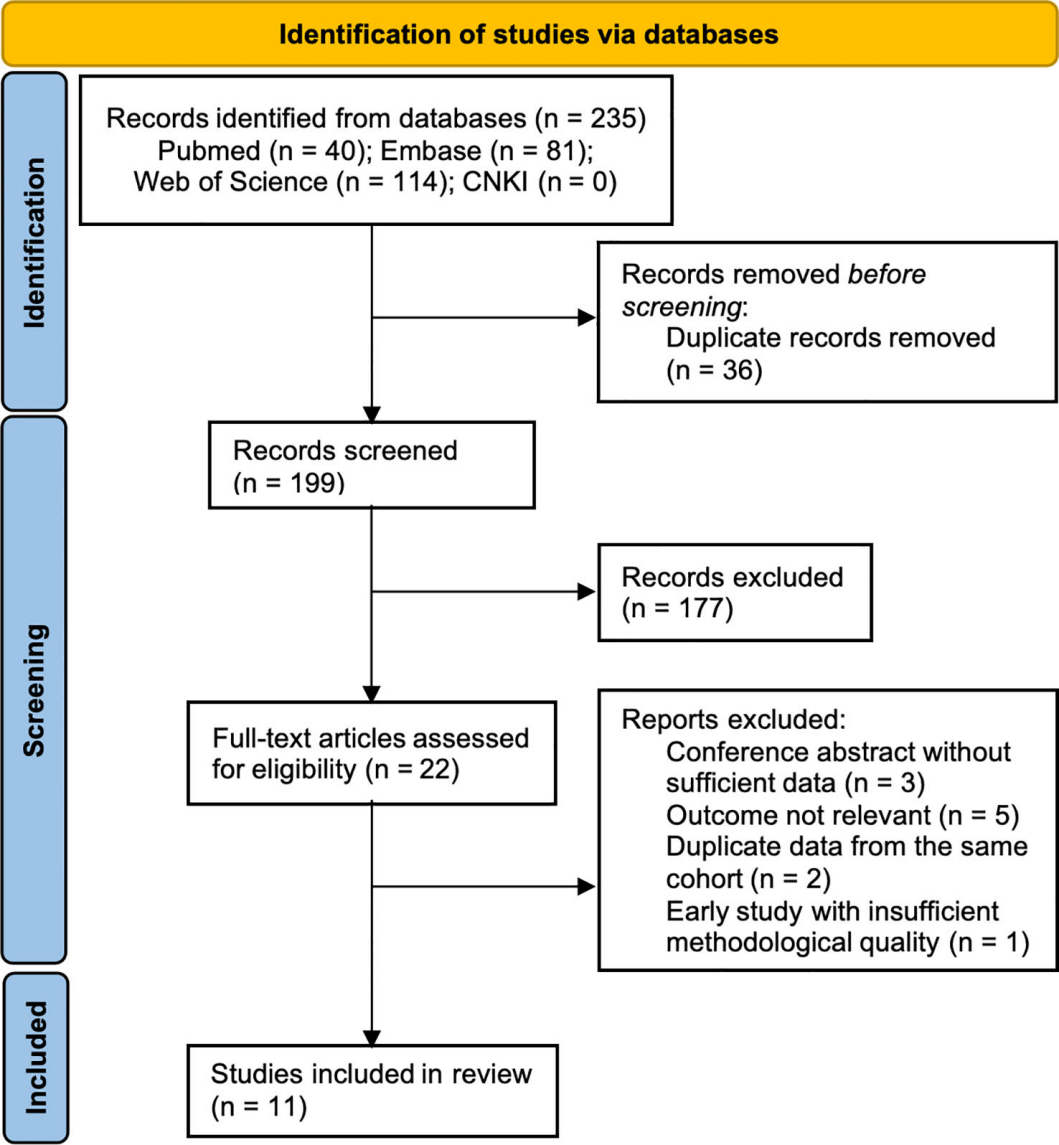
Flow chart of article selection.

Using both electronic database searches and manual screening, a total of 235 records were initially identified. After removing 36 duplicates, 199 unique records remained. Based on the titles and abstracts, 177 articles were excluded owing to their content not matching the review. The full texts of the remaining 22 articles were reviewed in detail. Of these, 11 were excluded for the following reasons: conference abstracts without sufficient data (n = 3), outcome not relevant (n = 5), duplicate data from the same cohort (n = 2), and early study with insufficient methodological quality (published in 1987, n = 1). Finally, 11 studies (14, 15, 19–27) met the inclusion criteria and were included in the meta-analysis (**Figure 1**).

### 3.2 Study characteristics and quality

We identified a total of 11 cohort studies from 4 countries (China, Austria, Germany, and Brazil) in the final analysis of the association between thyroid dysfunction and prognosis of liver failure, comprising 3595 participants. The sample sizes of the included studies ranged from 60 to 1862, and the methodological quality was moderate to high, with NOS scores ranging from 6 to 9. Most studies (n = 10) assessed mortality as the primary outcome, with follow-up periods ranging from 28 days to 2 years. One study additionally reported 28-day liver transplantation requirement. The baseline characteristics and quality assessment of the included studies are summarized in **Table 1**.

**Table 1:** Study characteristics and quality assessment of 11 cohort studies included in the meta-analysis.

Author, year	Country	Study design	Sample size (n)	Ages (years)	Male (%)	Type of liver failure	Thyroid dysfunction type	Outcomes	Key Findings	Limitations	NOS score
Chenjunfeng_2022 (15)	China	Retrospective cohort study	1862	Mean: NA	88.90%	HBV-ACLF	Low TSH	90-day mortality	Serum TSH independently predicts 30/90-day survival in HBV-ACLF and correlates negatively with MELD/CTP scores.	Short 90-day follow-up, single baseline TSH measurement, exclusion of 579 patients.	9
Yichen Wu_2014 (25)	China	Prospective cohort study	75	Mean: 42.8	90.7%	HBV-ACLF	Low TSH	1-year mortality	Serum TSH is an independent predictor of 1-year mortality in HBV-ACLF and correlates negatively with MELD score.	Small sample size (75 patients), limited confounder adjustment, and single-center design may restrict generalizability.	8
Zhangjian_2024 (26)	China	Retrospective cohort study	122	Mean: 45.6	82.0%	HBV-ACLF	Low FT3	90-day mortality	FT3 dynamic changes (3 types: persistent normal, continuous decrease, U-shaped) relate to HBV-ACLF severity and 90-day prognosis; single △FT3 and △FT3 range are independent predictors.	Small sample size (122 patients), single-center retrospective design.	7
Lin Lin_2024 (20)	China	Retrospective cohort study	81	NA	NA	HBV-DC with HE	Low FT3	1-year mortality	Low FT3 (not FT4/TSH) is an independent risk factor for 1-year mortality in HBV-DC with HE (n=81)	Single-center study with small sample size (n=81); partial retrospective data collection (selection bias).	8
Olympia Anastasiou_2015 (27)	Germany	Retrospective cohort study	84	Mean: 40.4	46.4%	ALF	Low TT3	28-day liver transplant requirement	In 84 acute liver failure (ALF) patients, higher TSH, total T3 (TT3) and total T4 (TT4) correlate with spontaneous recovery (SR); over 50% of patients have abnormal thyroid parameters (linked to poor outcomes), while free T3/fT4 show no intergroup differences; T3 fails to rescue acetaminophen-induced hepatocyte damage *in vitro*.	Small sample size (n=84) with limited statistical power and generalizability; retrospective study design, inherent to selection bias.	8
Lukas Hartl_2024 (22)	Austria	Ambispective cohort study	297	Median: 56.0	67.0%	ACLD	Low FT3	2-year mortality	In 297 ACLD patients, fT3 declines with disease progression; fT3/TSH independently correlate with CRP; lower fT3 is an independent predictor of decompensation, ACLF and liver-related death.	No assessment of thyroglobulin, reverse T3, intrahepatic deiodinase activity or thyroid hormone-binding globulin.	9
Nardin_2024 (24)	Brazil	Prospective cohort study	119	Mean: 53.5	71.4%	AD	Low FT3	90-day mortality	In 119 cirrhotic acute decompensation patients, lower fT3, higher fT4/TSH, and lower fT3/fT4 ratio correlate with severe liver disease (Child-Pugh C, ACLF); TSH, fT3/fT4 ratio, and MELD are independent predictors of 90-day mortality; the new MELD+TSH+fT3/fT4 model (AUROC = 0.899) outperforms individual indicators.	Small sample size (n=119), heterogeneous patient population; thyroid hormones measured only at admission (not generalizable to ICU/transplant settings); new prognostic model unvalidated.	9
Mona-May Langer_2023 (23)	Germany	Prospective cohort study	437	Mean: 54	57.2%	CC, AD, ACLF	Low FT3	90-day mortality	FT3 decreases with disease progression, TSH shows a U-shape; infections lower FT3 in AD; low FT3 independently predicts 90-day mortality.	Cross-sectional design with only baseline thyroid hormone measurements	8
Hong-Ling Feng_2020 (14)	China	Retrospective cohort study	73	Mean: 49.6	75.86%	ALF, SALF, ACLF	Low FT3	90-day mortality	FT3 negatively correlated with MELD score; the FT3-MELD model outperformed MELD, CTP, and CLIF-SOFA in predicting 3-month mortality.	Small sample size; single-center retrospective design.	8
Liuxing_2023 (21)	China	Retrospective cohort study	60	Mean: 44.4	83.3%	ALF, SALF, ACLF	Low FT3	180-day mortality	In 60 liver failure patients, low TT3 (78.2%) and FT3 (69.1%) were common, with higher incidence in non-survivors; non-survivors had higher MELD and lower TT3/FT3. MELD negatively correlated with TT3/FT3/TSH/respiratory quotient (RQ), RQ positively with TT3/FT3	Small sample size (n=60); no dynamic monitoring of thyroid hormones; unstable results of multivariate analysis.	6
Huangxiaoquan_2020 (19)	China	Retrospective cohort study	385	Mean: 56.6	50.91%	Cirrhosis with portal hypertension	Low FT3	2-year mortality	In 385 cirrhotic portal hypertension patients, low FT3 correlates with higher Child-Pugh/MELD scores, lower hemoglobin/albumin, and poorer 2-year survival; FT3 negatively associates with disease severity scores; FT3 and prothrombin time are independent prognostic factors.	Dominant etiology (hepatitis B) may limit generalizability to other causes; no prospective follow-up of thyroid function.	9

ACLD, advanced chronic liver disease; ACLF, acute-on-chronic liver failure; AD, acute decompensation; ALF, acute liver failure; CC, compensated cirrhosis; DC, decompensated cirrhosis; FT3, free triiodothyronine; HBV, hepatitis B virus; HE, hepatic encephalopathy; NA, not available; NOS, Newcastle–Ottawa Scale; SALF, subacute liver failure; TSH, thyroid-stimulating hormone; TT3, total triiodothyronine.

### 3.3 Overall meta-analysis

**Figure 2:**
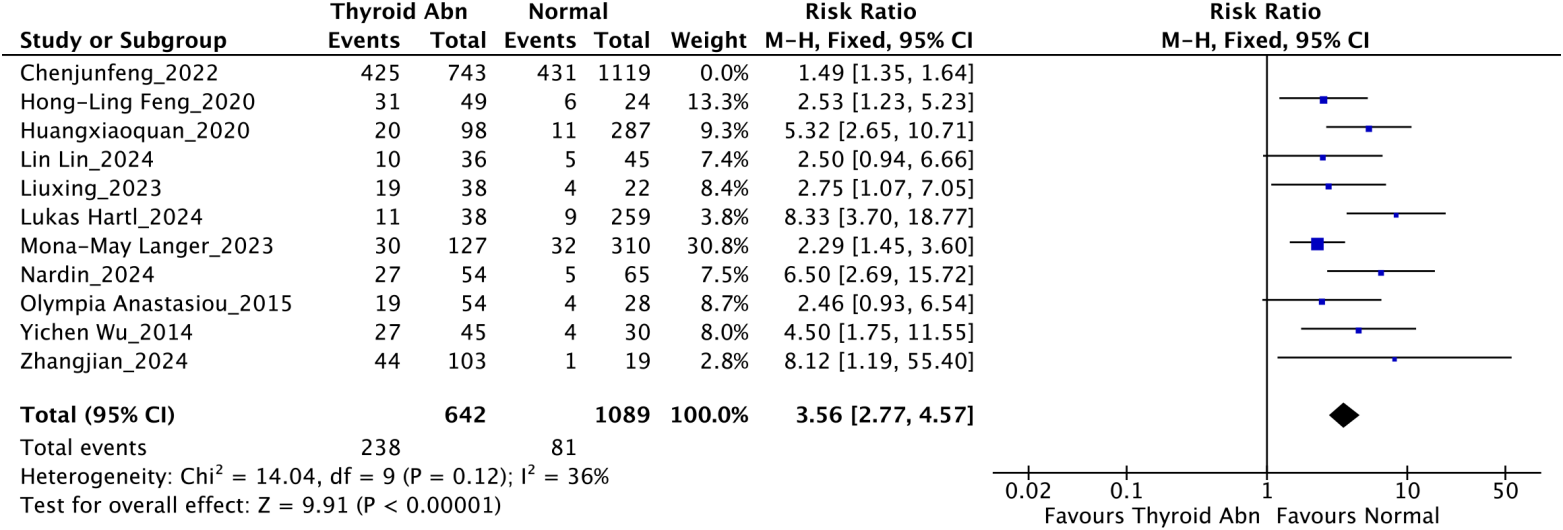
Pooled analysis of the association between thyroid dysfunction and mortality risk in liver failure patients, after excluding the study with low TSH definition.

As illustrated in **Figure 2**, the initial analysis showed substantial heterogeneity (*I²* = 82%). After excluding the study that defined thyroid dysfunction as low TSH, the overall meta-analysis of the remaining studies demonstrated that thyroid dysfunction was significantly associated with an increased risk of mortality in patients with liver failure (RR = 3.56, 95% CI: 2.77–4.57; *I²* = 36%, *P* < 0.00001). This exclusion was justified by both statistical and pathophysiological considerations: the excluded study’s TSH-based definition reflected central thyroid axis dysfunction, whereas the remaining studies used FT3-based definitions consistent with liver failure-induced NTIS, eliminating cross-phenotype heterogeneity.

**Figure 3:**
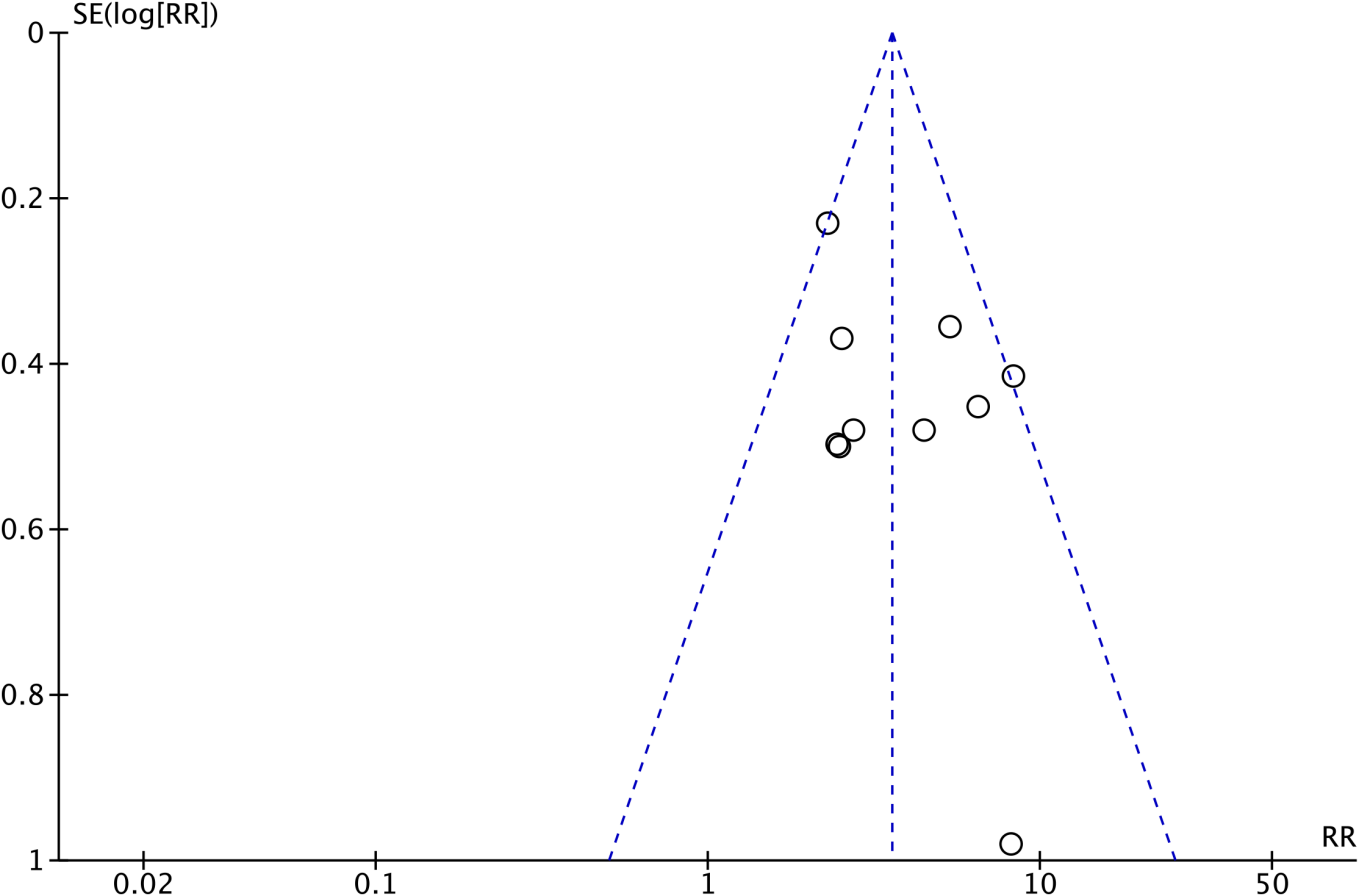
Funnel plot evaluating potential publication bias in the included studies. The plot demonstrates a visually symmetrical distribution of effect sizes, indicating a low likelihood of publication bias.

The funnel plot (**Figure 3**) showed a symmetrical distribution of effect sizes, suggesting no substantial publication bias. This was further supported by Begg’s test (*P* = 0.788) and Egger’s test (*P* = 0.188), indicating the robustness of the pooled results.

### 3.4 Subgroup analyses

**Figure 4:**
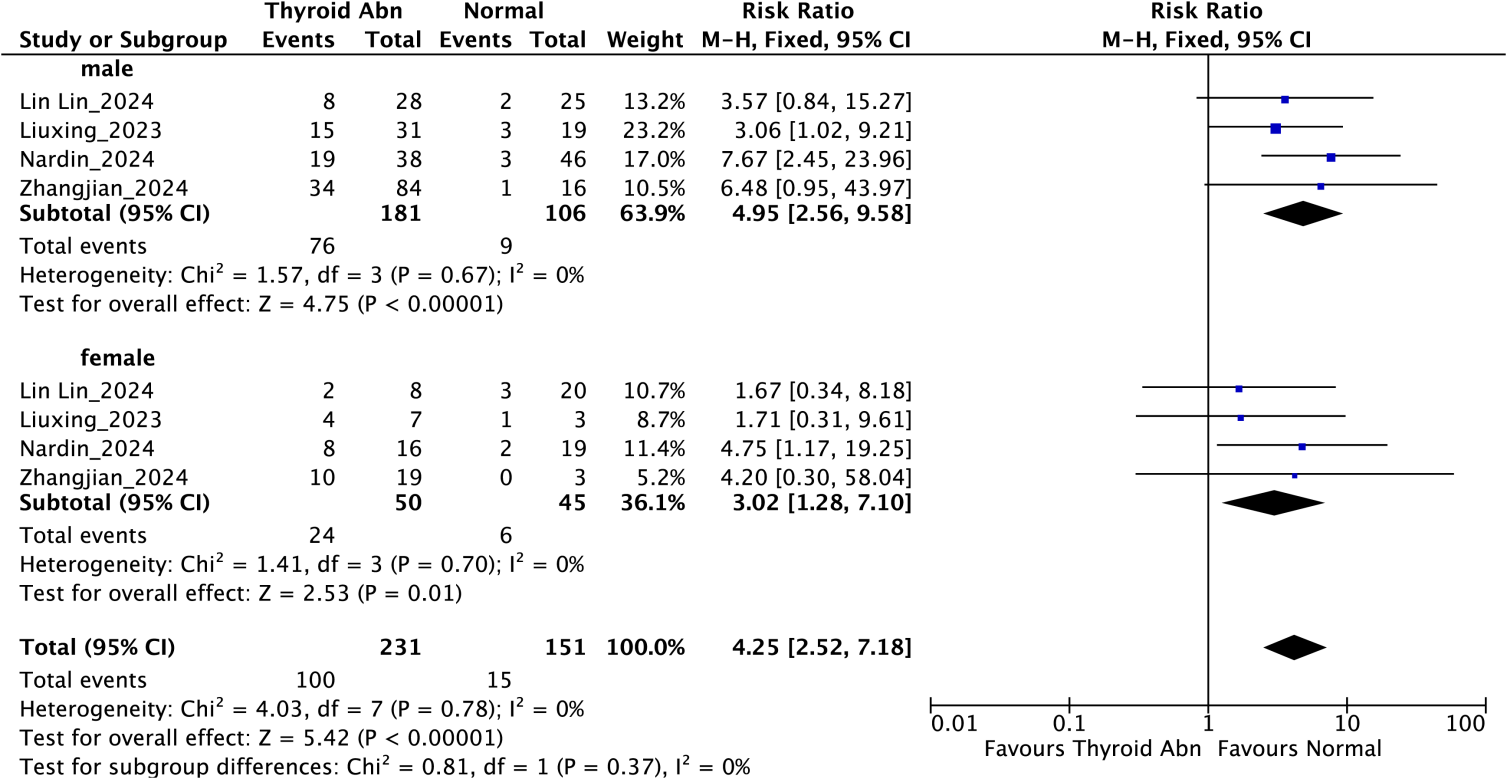
Subgroup analysis stratified by sex evaluating the association between thyroid dysfunction and mortality in patients with liver failure.

Subgroup analysis stratified by sex showed that thyroid dysfunction was significantly associated with higher mortality in both male and female individuals with liver failure (**Figure 4**). Among male patients, the combined RR reached 4.95 (95% CI: 2.56-9.58; *I²* = 0%, *P* < 0.00001), whereas in females it was 3.02 (95% CI: 1.28-7.10; *I²* = 0%, *P* = 0.01). The test for subgroup differences did not reveal significant heterogeneity between sexes (*P* for interaction = 0.37), suggesting a consistent association across genders.

**Figure 5:**
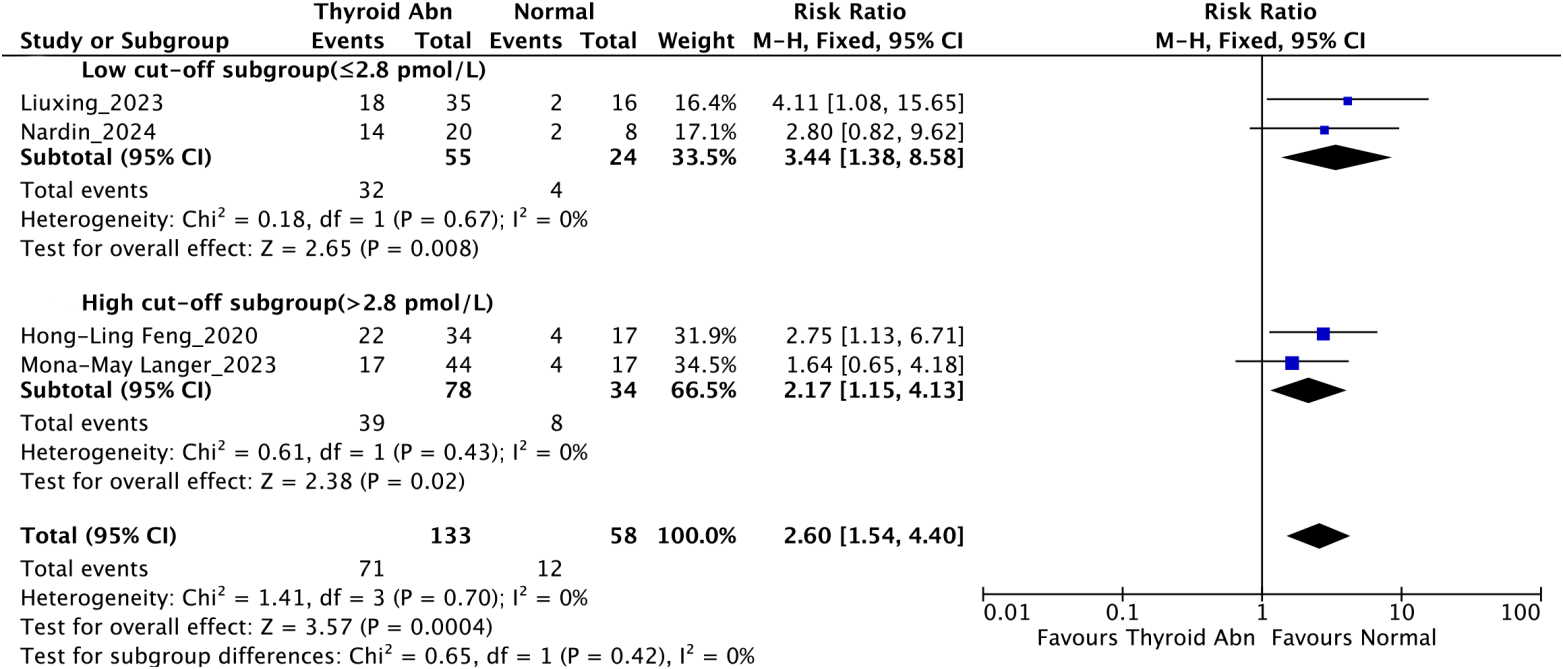
Subgroup analysis stratified by FT3 cut-off values evaluating the association between thyroid dysfunction and mortality in patients with liver failure.

Subgroup analysis based on FT3 cut-off values among patients with ACLF (**Figure 5**) revealed that low FT3 levels (≤ 2.8 pmol/L) were significantly associated with increased mortality (RR = 3.44, 95% CI: 1.38-8.58; *I²* = 0%; *P* = 0.008). Similarly, in patients with higher FT3 levels (> 2.8 pmol/L), thyroid dysfunction remained significantly correlated with mortality (RR = 2.17, 95% CI: 1.15-4.13; *I²* = 0%; *P* = 0.02). Between-subgroup comparison indicated no significant heterogeneity (*P* for interaction = 0.42).

**Figure 6:**
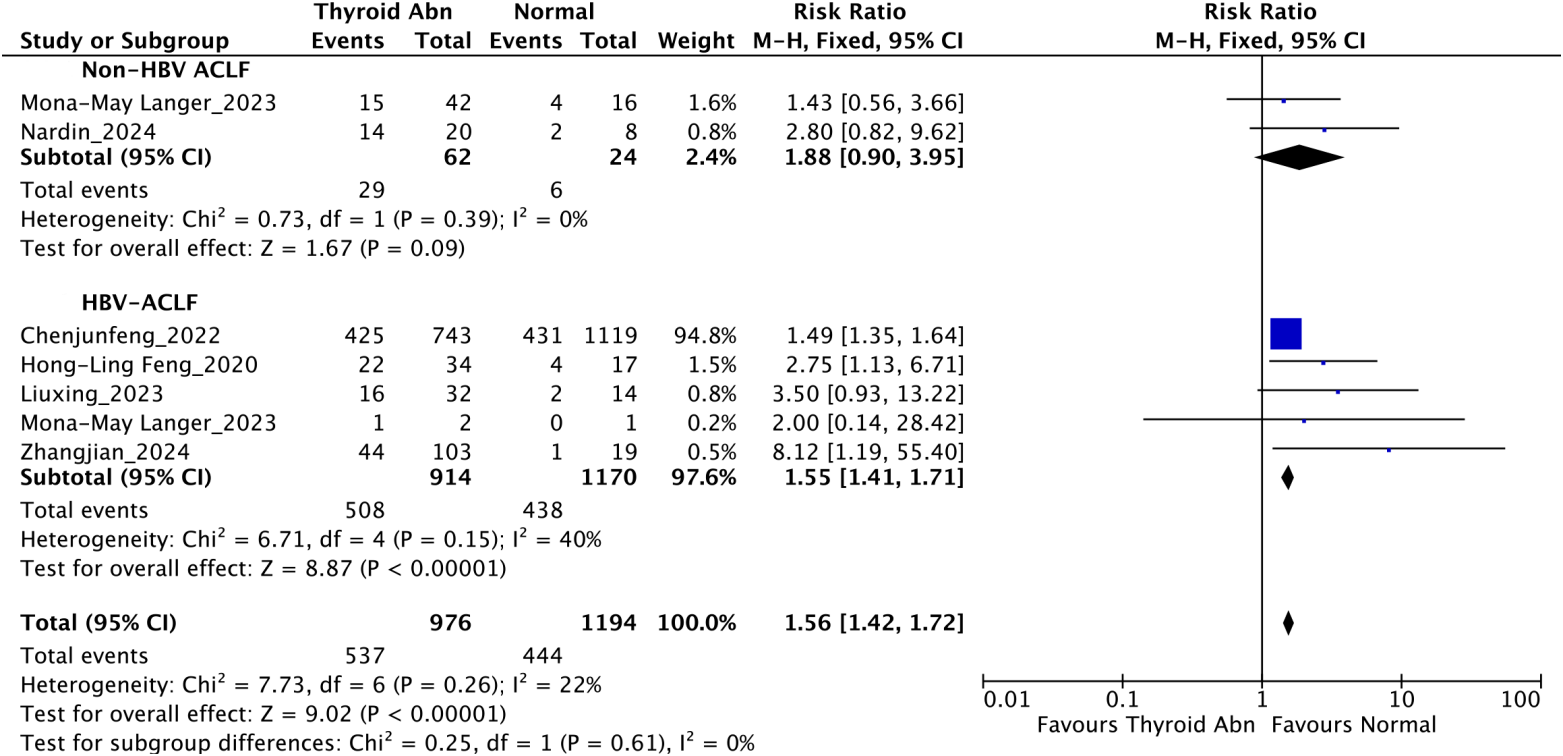
Subgroup analysis stratified by etiology evaluating the association between thyroid dysfunction and mortality in patients with liver failure.

Subgroup analysis by etiology (**Figure 6**) indicated that thyroid dysfunction was significantly linked to elevated mortality in HBV-ACLF patients (RR = 1.55, 95% CI: 1.41–1.71; *I²* = 40%; *P* < 0.00001). In contrast, among non-HBV-ACLF patients, this association did not reach statistical significance (RR = 1.88, 95% CI: 0.90–3.95; *I²* = 0%; *P* = 0.09). When data from all etiologies were combined, thyroid dysfunction continued to show a significant correlation with mortality (RR = 1.56, 95% CI: 1.42–1.72; *I²* = 22%; *P* < 0.00001). Although the point estimate suggested a potentially stronger association in the HBV-ACLF subgroup (RR = 1.55) compared to the non-HBV-ACLF subgroup (RR = 1.88), the test for subgroup differences was not statistically significant (*P* for interaction = 0.61), indicating that the effect of thyroid dysfunction may not differ substantially between these etiologies.

**Figure 7:**
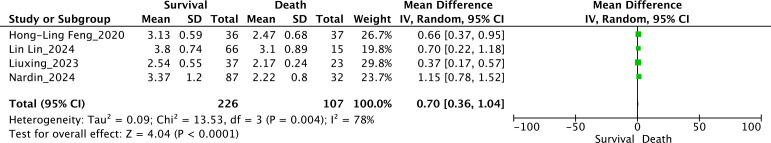
Subgroup analysis based on survival status comparing FT3 concentrations between survival and death groups in liver failure patients.​​ The overall pooled analysis demonstrates significantly lower FT3 levels in the death group compared to the survival group.

**Figure 8:**
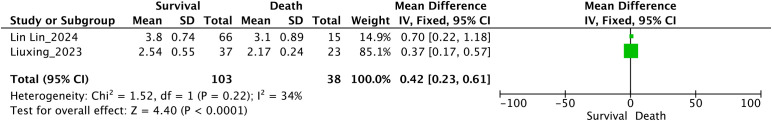
Subgroup analysis comparing FT3 levels between survival and short-term mortality groups in liver failure patients.​​ The pooled analysis demonstrates significantly lower FT3 levels in the short-term mortality group.

**Figure 9:**
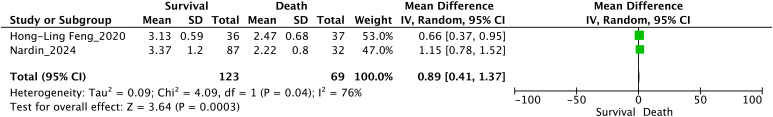
Subgroup analysis comparing FT3 concentrations between survival and long-term mortality groups in liver failure patients.​​ The pooled analysis shows a significant but smaller reduction in FT3 levels in the long-term mortality group compared to the survival group.

Subgroup analysis by survival status (**Figure 7**) showed a significant association between FT3 concentrations and mortality in liver failure patients. The overall analysis revealed that FT3 levels were significantly lower in the death group compared to the survival group (Total MD = 0.70 [0.36, 1.04]; *I²* = 78%; *P* < 0.0001). Further subgroup analysis (**Figure 8**) indicated that in the short-term mortality group, FT3 concentrations were significantly lower, with a mean difference of 0.89 [0.41, 1.37] (*I²* = 76%; *P* = 0.0003). In contrast, the long-term mortality group (**Figure 9**) showed a smaller but still significant difference in FT3 levels (MD = 0.42 [0.23, 0.61]; *I²* = 34%; *P* < 0.0001). These findings suggest that low FT3 levels are associated with both short-term and long-term mortality, with a stronger correlation in the short-term mortality group. Considerable heterogeneity was observed in both the overall analysis (*I²* = 78%) and the short-term mortality subgroup (*I²* = 76%). To explore potential sources of heterogeneity, we reviewed study characteristics including liver failure etiology, timing of FT3 measurement, measurement platforms, and patient severity at baseline. Several factors appear to contribute. For instance, Hong-Ling Feng_2020 included a mixed population consisting of acute liver failure, subacute liver failure, and acute-on-chronic liver failure, whereas Nardin_2024 focused exclusively on patients with acute decompensation. Variability in laboratory measurement platforms may also contribute to heterogeneity. Hong-Ling Feng_2020 used the Roche Cobas e411 electrochemiluminescence immunoassay, whereas Nardin_2024 measured thyroid hormones using the Siemens ADVIA Centaur XP chemiluminescent microparticle immunoassay. Taken together, the diversity in liver failure etiologies and the inconsistency in assay methods across studies are likely to account for part of the substantial heterogeneity observed in the survival vs. death comparisons.

### 3.5 Sensitivity analysis

To evaluate the robustness of the results, sensitivity analyses were conducted using both the leave-one-out approach and restriction to studies with higher methodological quality (NOS >7). As summarized in **Table 2**, omitting individual studies in turn yielded pooled RRs ranging from 3.01 to 3.58, indicating that no single study drove the overall effect. Notably, the excluding of *Chenjunfeng_2022* resulted in a substantial decline in heterogeneity, with *I²* decreasing from 82% to 36%, while the association with mortality remained statistically significant. When only high-quality studies were included, the summary estimate was comparable (RR = 3.30, 95% CI: 2.03–5.35), reinforcing the stability of the findings.

**Table 2:** Sensitivity analysis results.

Sensitivity analysis approach	RR (95% CI)	I² (%)	*P* value
Leave-one-out (all studies included)	3.36 (2.14-5.27)	82%	<0.00001
Excluding Chenjunfeng_2022	3.56 (2.77-4.57)	36%	0.12
Excluding Hong-Ling Feng_2020	3.49 (2.13-5.70)	83%	<0.00001
Excluding Huangxiaoquan_2020	3.16 (2.01-4.97)	80%	<0.00001
Excluding Lin Lin_2024	3.46 (2.14-5.60)	84%	<0.00001
Excluding Liuxing_2023	3.43 (2.12-5.55)	83%	<0.00001
Excluding Lukas Hartl_2024	3.01 (1.97-4.61)	78%	<0.00001
Excluding Mona-May Langer_2023	3.58 (2.10-6.12)	83%	<0.00001
Excluding Nardin_2024	3.13 (2.00-4.88)	80%	<0.00001
Excluding Olympia Anastasiou_2015	3.47 (2.14-5.61)	84%	<0.00001
Excluding Yichen Wu_2014	3.27 (2.05-5.20)	82%	<0.00001
Excluding Zhangjian_2024	3.24 (2.06-5.09)	83%	<0.00001
Excluding low-quality studies (NOS ≤7)	3.30 (2.03-5.35)	84%	<0.00001

## 4 Discussion

The aim of our meta-analysis was to evaluate the relationship between thyroid dysfunction and prognosis in patients with liver failure. We found that low FT3 was consistently associated with increased mortality, and the risk remained stable across subgroup analyses by sex, FT3 cut-off, and etiology. Sensitivity analyses further confirmed the robustness of this association. Notably, the exclusion of *Chenjunfeng_2022* substantially reduced heterogeneity, likely due to the distinct definition of thyroid dysfunction in this study (low TSH rather than low FT3), reflecting central rather than peripheral dysregulation. This distinction is not merely semantic: low TSH reflects potential central hypothalamic-pituitary dysfunction, which is different in pathophysiology from the NTIS caused by liver failure.

From a mechanistic perspective, reduced FT3 is a core feature of NTIS, may reflect the impaired hepatic clearance capacity and altered deiodinase activity under liver injury conditions (14, 28). These hormonal abnormalities may be linked to, rather than definitively cause, metabolic disorders, systemic inflammation, and immune dysfunction, thereby potentially contributing to multi-organ failure and increasing mortality death (29). Given the observational nature of the included studies, the biological explanations should be viewed as hypotheses rather than established mechanisms. Our subgroup analysis further revealed that the association between FT3 concentrations and mortality differed between short-term and long-term outcomes. FT3 levels were significantly lower in the death group compared to the survival group, particularly in the short-term mortality group. This finding suggests that FT3 might serve as a potential prognostic marker for liver failure short-term outcomes, potentially due to its role in rapidly modulating metabolic processes and inflammation during acute liver failure (14). It is critical to acknowledge that the observed association does not establish causality, and FT3 levels should not be interpreted as a direct causal determinant of mortality.

In the etiology subgroup analysis, a significant association was observed in HBV-ACLF patients, while the result in non-HBV-ACLF patients did not reach statistical significance. However, the test for subgroup differences was not significant (*P* for interaction = 0.61), indicating a lack of statistical evidence for a true etiological difference. Therefore, the observed numerical difference should be interpreted with caution. The non-significance in the non-HBV-ACLF subgroup is likely attributable to its smaller sample size and greater etiological heterogeneity. Although it is physiologically plausible that immune-mediated injury in HBV-ACLF could amplify the consequences of thyroid dysfunction (30, 31), our findings do not confirm this hypothesis. The results underscore the need for confirmation in larger, prospective cohorts with adequate power to detect true etiological interactions.

From a clinical perspective, the study demonstrates the feasibility of thyroid function evaluation in risk stratification for liver failure. Monitoring FT3 and other thyroid parameters may help identify patients at high risk of liver failure who could benefit from intensified surveillance or tailored interventions (20, 22). Clinically, measuring thyroid hormones is inexpensive, widely available, and can be incorporated into routine laboratory panels, which supports its potential use in early risk flagging. However, its application has important limitations: thyroid abnormalities in liver failure are highly dynamic, influenced by non-thyroidal illness, and may not reflect intrinsic thyroid gland dysfunction. The observation that FT3 concentrations were more strongly associated with short-term mortality highlights the potential for using FT3 as an early biomarker for predicting acute liver failure outcomes. Nonetheless, the absence of interventional evidence means that correcting low FT3 cannot currently be recommended as a therapeutic strategy. Whether thyroid hormone supplementation improves prognosis remains unknown, and any clinical applications should rely on future randomized trials (15).

Future research should focus on large-scale prospective cohort studies to validate the prognostic value of FT3 and other thyroid parameters in diverse liver failure populations. Mechanistic studies are also needed to clarify how thyroid hormone alterations contribute to systemic inflammation, metabolic dysfunction, and multiorgan failure. In addition, whether therapeutic modulation of thyroid hormones can improve clinical outcomes warrants rigorous evaluation through randomized controlled trials. The development of etiology-specific prognostic models incorporating thyroid function markers may further enhance individualized risk stratification in patients with liver failure.

Some limitations of this analysis need to be addressed. Primary, differences in study design, patient populations, and definitions of thyroid dysfunction may have influenced the pooled estimates. Additionally, the predominance of retrospective studies introduces potential selection and reporting biases. Our literature search focused on PubMed, Embase, Web of Science, and CNKI, excluding the Cochrane Library, Google Scholar, and other study registries. This may have resulted in the omission of eligible studies, particularly gray literature or unpublished data, thereby contributing to potential selection bias. Notably, we didn’t register a protocol with PROSPERO (International Prospective Register of Systematic Reviews) or INPLASY (International Platform of Registered Systematic Review and Meta-analysis Protocols) beforehand, which may lead to publication bias or duplication of efforts. Furthermore, subgroup analyses were unable to thoroughly investigate potential effect modifiers such as age, liver failure severity, or regional differences. Lastly, the observational nature of the included studies precludes causal conclusions, and residual confounding cannot be excluded.

## 5 Conclusion

In conclusion, our findings confirm that thyroid dysfunction, particularly low FT3 levels, is a significant prognostic factor linked to increased mortality in patients with liver failure. Subgroup analysis further revealed that FT3 concentrations were more strongly associated with short-term mortality compared to long-term mortality, suggesting its potential as an early biomarker for predicting acute liver failure outcomes. These results highlight the potential benefit of incorporating thyroid function assessment into routine clinical practice for improved risk stratification. However, further large-scale prospective studies are needed to establish causality and explore the therapeutic potential of addressing thyroid dysfunction.

## Data Availability

The original contributions presented in the study are included in the article/**Supplementary Material**. Further inquiries can be directed to the corresponding author.
