# Supplementary material for: Association between thyroid dysfunction and prognosis in patients with liver failure: a systematic review and meta-analysis
Source: Front Endocrinol (Lausanne). 2026 Jan 9;16:1748907. doi: 10.3389/fendo.2025.1748907 (PMC12827069; doi:10.3389/fendo.2025.1748907)
Supplement: Supplementary file 1 [file DataSheet1.pdf]

1. Strategy for PubMed, DATE: 2025/8/1

(
("liver failure"[Mesh] OR "hepatic failure"[tiab] OR "ACLF"[tiab] OR "acute-on-chronic liver failure"[tiab])
AND
("thyroid dysfunction"[Mesh] OR "hypothyroidism"[tiab] OR "hyperthyroidism"[tiab] OR "low T3 syndrome"[tiab] OR "TSH"[tiab] OR "free thyroxine"[tiab] OR "FT3"[tiab])
)
AND
("risk factors"[Mesh] OR "prognosis"[Mesh] OR "predictive value of tests"[Mesh] OR "mortality"[sh] OR "severity of illness index"[Mesh] OR "MELD score"[tiab])
NOT
("animals"[Mesh] NOT "humans"[Mesh])

2. Strategy for Embase, DATE: 2025/8/1

```
(
  (
    'thyroid dysfunction':ti,ab OR
    'hypothyroidism':ti,ab OR
    'low t3 syndrome':ti,ab OR
    'non-thyroidal illness':ti,ab OR
    ('tsh':ti,ab AND ('abnormal':ti,ab OR 'elevated':ti,ab OR 'decreased':ti,ab))
  )
  AND
  (
    'liver failure'/exp OR
    'acute-on-chronic liver failure':ti,ab OR
    'decompensated cirrhosis':ti,ab OR
    (('hepatic insufficiency'/exp) AND ('prognosis'/exp OR 'mortality'/exp))
  )
  AND
  (
    'risk factor':ti,ab OR
    'prognostic factor':ti,ab OR
```

'disease progression':ti,ab OR

'mortality':ti,ab

)

)

NOT ('immunotherapy'/exp OR 'bevacizumab'/exp OR 'pd-1 inhibitor':ti,ab)

NOT ('carcinoma'/exp OR 'cancer':ti,ab)

3. Strategy for WOS, DATE: 2025/8/1

(TS=("thyroid dysfunction" OR "hypothyroidism" OR "hyperthyroidism" OR

"low T3 syndrome" OR "euthyroid sick syndrome" OR "non-thyroidal illness" OR

("TSH" NEAR/3 ("abnormal" OR "elevated" OR "decreased")) OR

("FT3" NEAR/3 ("low" OR "deficient" OR "decreased"))))

AND

(TS=("liver failure" OR "hepatic failure" OR "acute-on-chronic liver failure" OR "ACLF" OR

"decompensated cirrhosis" OR "end-stage liver disease" OR

("MELD score" OR "Child-Pugh score" OR "hepatic encephalopathy" OR "ascites")))

AND

(TS=("prognosis" OR "mortality" OR "risk factor" OR "predictive value" OR

"disease progression" OR "survival rate" OR "clinical outcome"))

NOT

(TS=("cancer" OR "carcinoma" OR "immunotherapy" OR "bevacizumab" OR "sorafenib" OR "atezolizumab"))

4. Strategy for CNKI, DATE: 2025/8/1

(KY = (Thyroid dysfunction + Hyperthyroidism + Hypothyroidism + Subclinical Hypothyroidism + Subclinical Hyperthyroidism + Low T3 Syndrome + Thyroid hormone abnormalities + TSH + FT3 + FT4))

AB = (Thyroid function + Thyroid hormones + TSH + FT3 + FT4))

(KY = (Liver failure + Hepatic failure + Acute-on-chronic liver failure + ACLF + Decompensated cirrhosis + Hepatic encephalopathy))

AB = (Liver failure + Hepatic failure + ACLF + Decompensated cirrhosis +

Hepatic encephalopathy))

(AB = (Prospective + Cohort + Follow-up + Prognosis))

KY = (Cohort study + Prospective study))

(SU = (Children + Pediatrics + Infants + Pregnancy + Tumor))

AB = (Children + Pediatrics + Infants + Pregnancy))

Newcastle-Ottawa Scale (NOS)

Total score: 9 points, evaluated across three main dimensions:

1. Selection (Up to 4 points)

- Representativeness of the exposed cohort
- Selection of the non-exposed cohort
- Ascertainment of exposure
- Outcome of interest not present at the start of the study

2. Comparability (Up to 2 points)

- Control of confounding factors (e.g., adjustment for age, gender, comorbidities, etc.)

3. Outcome (Up to 3 points)

- Adequacy of outcome assessment (e.g., confirmation of death from a registration system)
- Length of follow-up (≥28 days or 90 days)
- Completeness of follow-up (e.g., reporting of loss to follow-up rate)
